# Desmoplastic Small Round Cell Tumor as a Rare Cause of an Incarcerated Epigastric Linea Alba Hernia: A Case Report and Literature Review

**DOI:** 10.7759/cureus.61729

**Published:** 2024-06-05

**Authors:** Matej Pekař, Zdeněk Janda, Kristýna Franková

**Affiliations:** 1 Department of Vascular Surgery, Hospital AGEL Třinec-Podlesí, Třinec, CZE; 2 Department of Physiology, Faculty of Medicine, Masaryk University, Brno, CZE; 3 Department of Surgery, Hospital AGEL Ostrava-Vítkovice, Ostrava, CZE; 4 Department of Pathology, Hospital AGEL Nový Jičín, Nový Jičín, CZE

**Keywords:** sarcoma, metastasis, hernia, incarcerated, desmoplastic small round cell tumor

## Abstract

Desmoplastic small round cell tumor is a very rare soft tissue sarcoma with a bleak prognosis and short patient survival. The most common occurrence is in 20-30-year-old men. Our study presents the case report of a 40-year-old patient who was diagnosed with this sarcoma. The first symptom of the illness was an incarcerated epigastric hernia with sarcoma metastasis resembling an intestinal loop in an ultrasound image. The fluorescence in situ hybridization (FISH) method showed a fusion of the *EWS* and *WT1* genes. Systemic palliative chemotherapy using the VDC-IE (vincristine, doxorubicin, cyclophosphamide, ifosfamide, and etoposide) regimen was chosen instead of further surgery due to the disease's generalization. However, the therapy failed to halt the disease progression and was thus terminated after 18 months. The patient's overall survival was 19 months. The rare character of this disease complicates the diagnostics in clinical practice. Nevertheless, rare sarcomas should be considered in patients with non-specific abdominal symptoms, including patients with incarcerated ventral hernia.

## Introduction

Desmoplastic small round cell tumor (DSRCT) is a rare malignant neoplasm with a very aggressive behavior. It was first described in 1989 by William L. Gerald and Juan Rosai, who then formally named it in 1991 [1]. The incidence of DSRCT ranges from 0.2 to 0.74 cases per million people per year [2-4], and it most commonly affects young adults aged 20-30 [5], with a male-to-female predominance of 4:1 [6]. The method of how to visualize and map the genetic material in an individual's cells including specific genes or portions of genes to diagnose the chromosomal abnormalities and other genetic mutations is called fluorescence in situ hybridization (FISH). On the molecular level, the diagnosis is established by observing a reciprocal balanced chromosome translocation t(11;22) (p13;q12) that leads to a fusion of the EWS and WT1 genes, producing a chimeric protein with transcriptional regulatory activity [7]. The chimeric transcription factor regulates the expression of specific target genes, such as growth factor receptor, platelet-derived growth factor, receptor for insulin-like growth factor, and receptor for epidermal growth factor [8]. The dysregulation leads to tissue differentiation, proliferation, and adhesion and metastasis of tumor cells. DSRCT most often originates on the surface of the peritoneum in the abdominal and pelvic cavity and has already spread widely when discovered [9]. In rare cases, the primary tumor develops extra-abdominally in the kidney, ovary, testes, cervical lymph node, liver, parotid gland, and submaxillary gland, intraspinally or subcutaneously [10]. There are no known risk factors related to the development of this disease, and the pathogenesis remains unclear [11].

DSRCT is usually underdiagnosed as it often manifests itself only with non-specific symptoms depending on the affected area. For the most common intra-abdominal localization, the presentation includes non-specific dull abdominal pains and disorders of the intestinal passage. However, DSRCT can be asymptomatic for a long period, and the symptoms usually present at the late stage. In a few cases, there was described DSRCT with umbilical or inguinal hernia. We report a very unusual initial manifestation of the sarcoma as an incarcerated epigastric ventral hernia.

## Case presentation

The patient, who first presented in 2023 [12], was a 40-year-old male, suffering from gastroesophageal reflux disease on proton pump inhibitor therapy with no history of abdominal surgery. He was treated in our surgical outpatient clinic for a painful resistance in the abdominal wall. He stated that he had noticed a bulge in his abdomen wall for a long time. Now, he felt stiffness and pain in the area of resistance for two months, especially when sneezing. He had no intestinal tract disorder and no other symptoms, and he was without weight loss. Clinically, there was a palpable immobile hard resistance characteristic of an incarcerated epigastric linea alba hernia, measuring 8×6×5 cm. On palpation, the resistance was painful, and the rest of the abdomen was normally palpable, soft, painless, and without peritoneal irritation. Based on the clinical investigation, we suspected an incarcerated hernia. However, the laboratory findings revealed normal levels of leukocytes and C-reactive protein, and the abdominal X-ray showed no abnormalities. We followed with the ultrasound of the abdomen, which described an incarcerated hernia with contents resembling a thin loop with free fluid and a hypoechogenic mass of unclear etiology, leading to a referral of the patient to our department for an urgent surgical revision. There was no preoperative CT scan performed.

**Figure 1:**
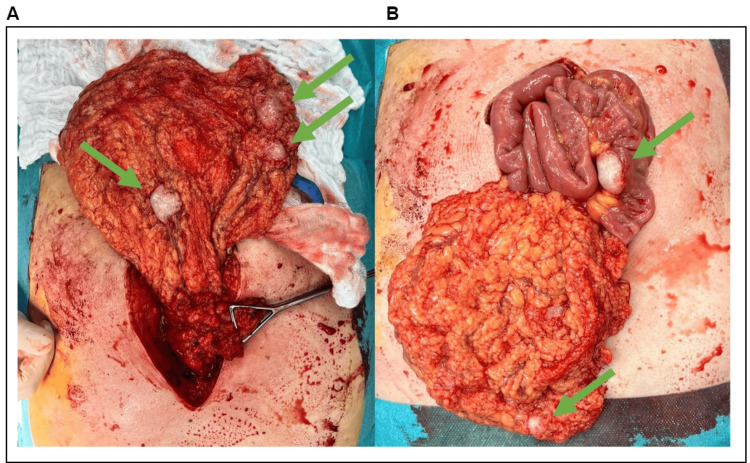
Intraoperative finding (A) The laparotomy with anteposed omentum with white ovoid metastases (green arrows). (B) The laparotomy with anteposed omentum and thin bowel loop covered with metastases (green arrows).

A hernia sac was dissected intraoperatively with a surprising finding after its opening. The content of the hernia sac was the incarcerated omentum, which had a white elastic resistance of ovoid shape, size 3×2×2 cm. A metastatic tumor process was suspected. After cutting the hernia gate, it was decided to enlarge the surgical wound in the sense of an exploratory laparotomy. The entire omentum was covered with white metastases of 2 cm in size (Figure 1), and there were also metastases of 1.5 cm in size all over the retroperitoneum, in the pelvic cavity, on the liver, and on the peritoneum of the anterior abdominal wall. A larger tumorous mass was located on the mesoappendix and part of the ascending colon. The intestine was collapsed, and peristalsis was present, without signs of stenotic lumen of the intestine. Numerous resistances were also palpable in the liver. We performed a resection of the encrusted part of the omentum with metastases followed by a biopsy from another deposit intra-abdominally. The biopsy specimens were sent for histopathological examination. No further surgical intervention was indicated for the generalization of the disease.

**Figure 2:**
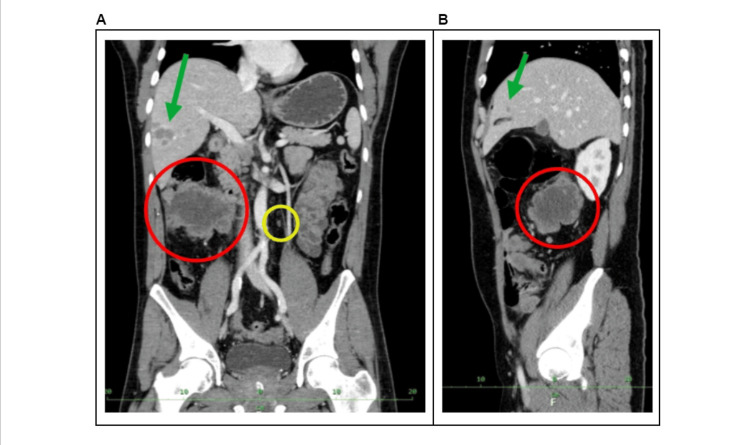
Staging CT examination (venous phase) (A) Anteroposterior reconstruction with the largest tumorous mass situated in the area of the ascending colon (red circle), paraaortal malignant lymph node (yellow circle), and liver metastasis (green arrow). (B) Lateral reconstruction of the abdomen with liver metastasis (green arrow) and tumorous mass in the area of the ascending colon (red circle).

Postoperatively, the patient had standard wound healing and was discharged to outpatient care on the seventh postoperative day in a clinically stable condition. The results of the staging CT scan were consistent with the intraoperative findings of multiple metastases in the omentum, liver, and peritoneum and a massive tumor in the ascending colon (Figure 2).

**Figure 3:**
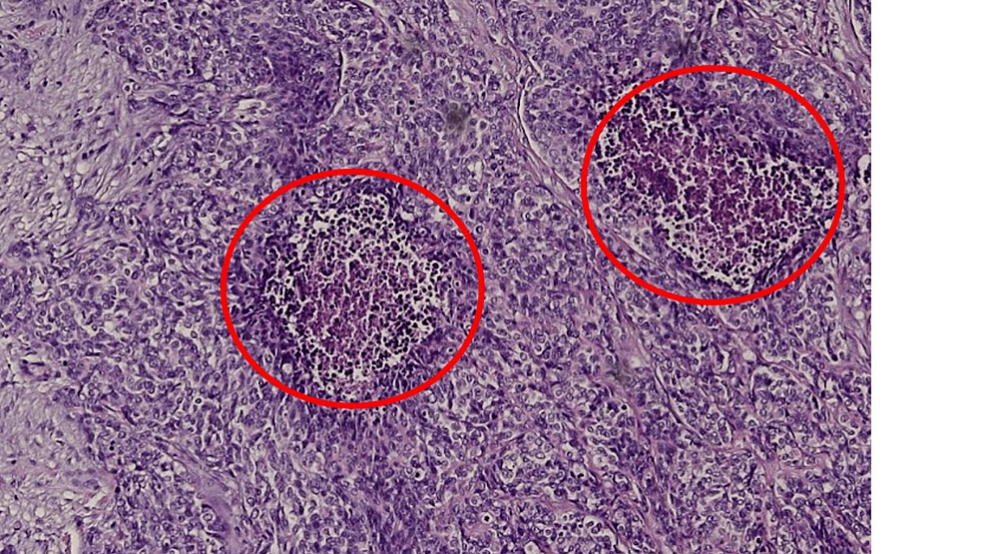
Histological sample Hematoxylin/eosin, captured tumor structures with desmoplastic stroma, small hyperchromic cells with blue nuclei with only rarely visible nucleoli. Necrotic cells are highlighted by red circles.

However, negativity of the oncomarkers Ca19-9 and CEA did not support the clinical assumption of a generalized colorectal tumor. Histology showed that it was a rare type of soft tissue sarcoma (Figure 3), and the FISH method demonstrated the rearrangement of the EWS-WT1 gene which, together with the tumor morphology and immunophenotype, established the diagnosis of DSRCT.

After consultation with the sarcoma center, the patient was referred to an oncologist. Due to the extent of the disease with a poor prognosis and a typical survival of about 1-1.5 years, palliative chemotherapy was indicated, followed by restaging after three months and further therapy depending on response. Systemic chemotherapy (CHT) was planned in the VDC-IE (vincristine, doxorubicin, cyclophosphamide, ifosfamide, and etoposide) regimen, alternating every two weeks, a combination also used in the treatment of other sarcomas (e.g., Ewing's sarcoma).

**Figure 4:**
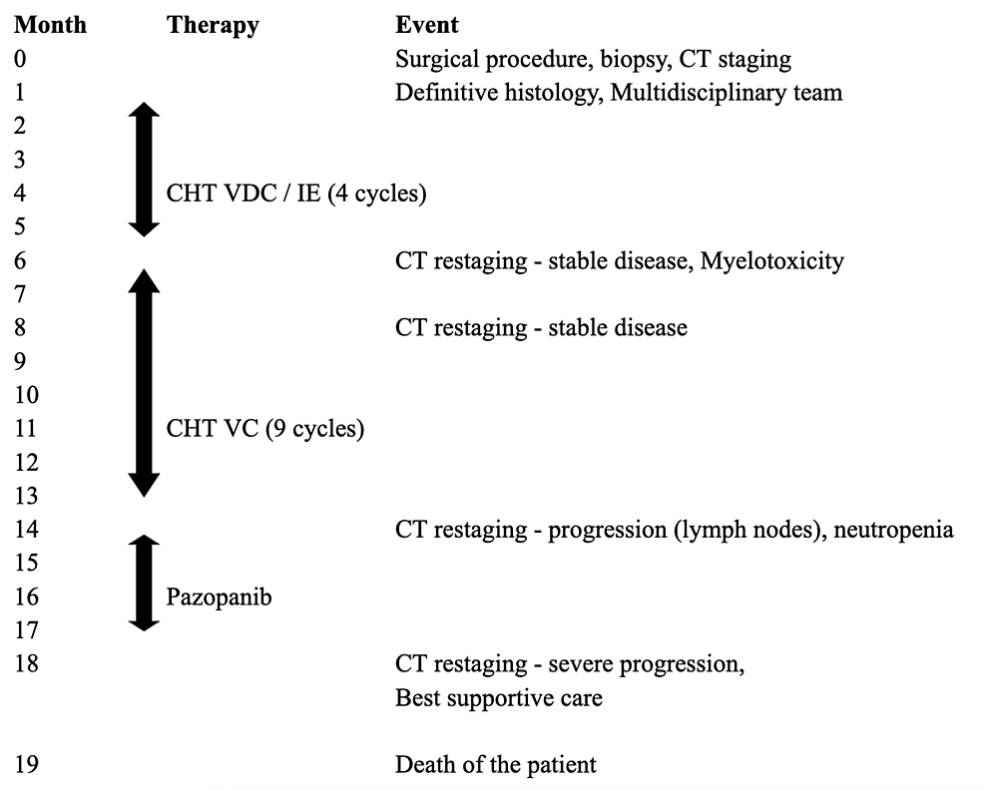
Flowchart of the therapy The flowchart depicts the timeline from the first medical contact to the death of the patient. Black arrows represent the pharmaceutical therapy intervals. CHT: chemotherapy; VDC/IE: vincristine, doxorubicin, cyclophosphamide, ifosfamide, and etoposide

**Figure 5:**
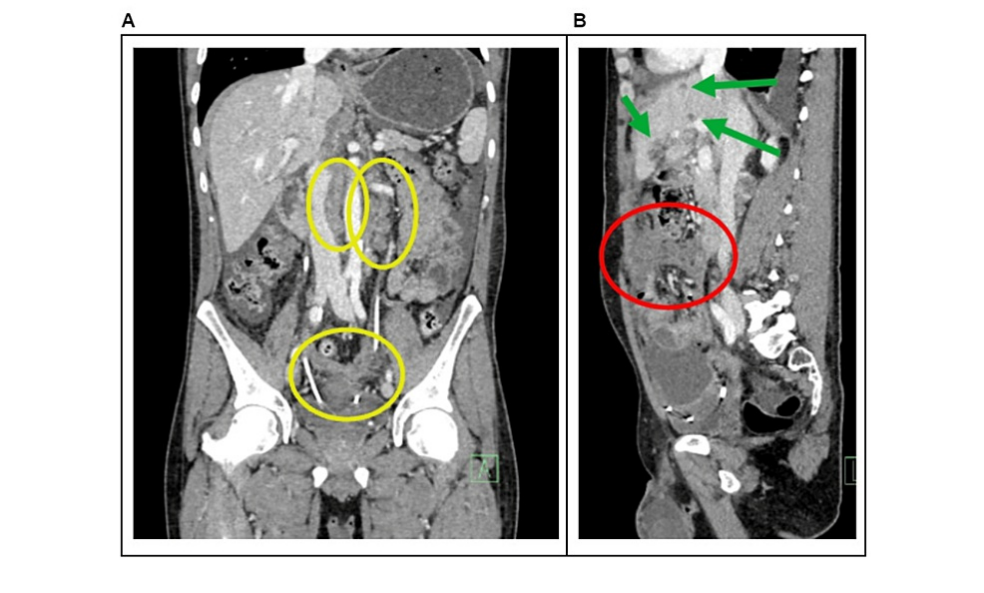
Restaging CT examination at 18 months (venous phase) (A) Anteroposterior reconstruction with the progression of paraaortal malignant lymph nodes (yellow circles). (B) Lateral reconstruction of the abdomen with liver metastases (green arrows) and tumorous mass in the area of the ascending colon (red circle).

Palliative CHT started two months after the first biopsy (Figure 4) via a PICC vascular port. According to the schedule, CHT was always administered during hospitalization. In total, the patient received five cycles of VDC and four cycles of IE and was subsequently hospitalized for myelotoxicity (with anemia, neutropenia, and thrombocytopenia). CHT was continued after restaging CT, which showed stable disease, with another nine cycles of VC (no doxorubicin, no IE for myelotoxicity). After a year of CHT application, it was discontinued due to persistent neutropenia and new pathological nodules on CT. Subsequently, the patient was treated with pazopanib until disease progression. The patient was diagnosed with nephrolithiasis as an intercurrent during oncology therapy, and bilateral JJ stents were urgently inserted with one elective replacement. During the insertion of JJ stents, the patient aspired and developed a mild pneumonia, which was treated with a double combination of antibiotics (cefotaxime and metronidazole). The patient's follow-up was limited to repeated spasmodic abdominal pain and a slight weight loss during the entire follow-up period without any passage disorders. Eighteen months after the diagnosis, the oncological therapy was terminated due to the progression of findings on the CT restaging: metastases in the S3 segment of the lungs, progression of metastases spread in the peritoneum, progression of the number and size of liver metastases, and dilatation of the left renal pelvicalyceal system despite the JJ stent (Figure 5). The oncologist referred the patient to mobile hospice care for the best supportive care. His anesthesia was achieved by an epidural catheter and opiate patches. The patient's overall survival was 19 months. 

## Discussion

Sarcomas are a diverse group of malignant tumors that arise from connective tissue. They originate from mesenchymal cells that normally differentiate into muscle, fat, vascular, bone, fibrous, or cartilaginous cells. The most common sarcomas arise from soft tissue (liposarcoma, rhabdomyosarcoma, leiomyosarcoma, fibrosarcoma, and others), while the rarer forms arise from bone (osteosarcoma, chondrosarcoma, Ewing's sarcoma) [13]. DSRCT is a rare subset of soft tissue sarcomas.

Therapeutic options include surgical resection, CHT, radiotherapy, and targeted therapy. However, complete surgical resection is usually not possible due to the presence of multiple neoplastic nodules and the strong ability of the tumor to metastasize. For larger tumors, the resection is usually performed after 3-4 cycles of CHT. In some cases, the peritoneal tumor masses are removed using the tangential dissection technique. Another important role of surgery is to relieve some symptoms, such as intestinal obstruction, which occurs in up to half of patients. Successful resection is followed by adjuvant radiotherapy with an external dose of 30 Gy to the abdomen and pelvic cavity. CHT is recommended for all patients, including advanced and inoperable stages, as well as in palliative therapy, because the tumor shows high chemosensitivity, even if only with a short-lasting effect. A specific protocol is not recommended, and CHT drugs such as vincristine, doxorubicin, cyclophosphamide, ifosfamide, and etoposide are used. Another therapy option is hyperthermic intraperitoneal chemotherapy (HIPEC) using doxorubicin and cisplatin. HIPEC is considered for extensive intra-abdominal metastases, but its role remains controversial [14]. Targeted therapies include the use of a platelet-derived growth factor inhibitor (pazopanib, sunitinib, and others), an androgen receptor inhibitor (e.g., enzalutamide), and antibodies against the immunomodulatory molecule B7H3 (enoblituzumab) [11].

Average patient survival ranges from 1.5 to 2.5 years, and 15% survive for more than five years. The combination of successful surgical resection with adjuvant CHT achieves three-year survival in 58% of cases [15]. Other authors report a median survival of 34 months for operable and 14 months for inoperable findings [16].

Common features with the described behavior of DSRCT can be found in the presented case report. The patient was a young man who had been asymptomatic for a long time. This sarcoma was an intra-abdominal form with multiple metastases without a bowel passage disorder. However, its first manifestation as an incarcerated ventral hernia was highly unusual. Only a few similar cases were reported in the literature. We found three cases of inguinal or scrotal hernia and the study of 32 patients suffering from DSRCT, where umbilical hernia was presented in only four patients and no ventral hernia in the epigastric area was described [17-20] (Table 1).

**Table 1:** Details of cases of DSRCT with hernia reported in the literature DSRCT: desmoplastic small round cell tumor; A: antibody therapy; AWD: alive with disease; AWR: alive with remission; CHT: chemotherapy; DOD: dead of disease; mo: months; RT: radiotherapy; S: surgery resection

Publication	Gender	Age (years)	Location	Treatment	Follow-up
Ibilibor et al. [17]	Male	35	Scrotal hernia	S, CHT, RT	AWD, 19 mo
Kruger et al. [18]	Male	2	Inguinal hernia	CHT, S, A, RT	AWR, 34 mo
Sedig et al. [19]	Male	14	Inguinal hernia	S, CHT, RT	AWR, 24 mo
Lae et al. [20]	Male	32	Umbilical hernia	S, CHT, RT	DOD, 30 mo
Lae et al. [20]	Male	14	Incarcerated umbilical hernia	S	DOD, 10 mo
Lae et al. [20]	Male	15	Umbilical hernia	S, CHT	DOD, 16 mo
Lae et al. [20]	Male	30	Umbilical hernia	CHT	DOD, 17 mo

The finding was so surprising that even to an experienced radiologist, the ultrasound image of the contents of the hernia sac resembled an incarcerated thin loop, while intraoperatively an omentum with a metastasis was found. Spontaneous reposition of a thin loop may have occurred during the induction of anesthesia, but the induction was intentionally performed without myorelaxation, and all intestinal loops were vital during revision. For patients with indeterminate abdominal disorders, it is necessary to perform a diagnostic ultrasound and, in case of uncertainty, proceed to CT or MRI. Surgery could still be preceded by PET-CT when non-specific nodules are found, but this was not our case because the patient was indicated for surgical revision, and exploratory laparotomy should also be considered in unclear cases.

Histologically, tumor structures with a desmoplastic stroma interspersed with bands and spindles of small hyperchromic cells with blue nuclei, in which nucleoli were only rarely visible, with focal necrosis and spindles in the center of the tumor (Figure 3). Lymphovascular propagation of the tumor was found in several places. The rearrangement of the EWS-WT1 gene has been proven to accurately diagnose the disease. There are no known risk factors or specific oncomarkers for this disease, and DSRCT is not suitable for general screening. Due to the rapid spread of the metastases, the chance of its detection in the initial stages with the possibility of R0 resection is very small. All this contributes to short median patient survival.

## Conclusions

DSRCT is a rare soft tissue sarcoma that is not well-known in routine medical practice due to its low incidence. The tumor develops more often in younger men. Symptoms of the disease tend to be non-specific, and sarcoma is often underdiagnosed, which contributes to the short survival of patients with this tumor. The fusion of EWS and WT1 is characteristic for DSRCT, and systemic CHT is often used because the tumor is chemosensitive. We should consider the possibility of rare sarcomas in patients with non-specific abdominal symptoms. We should perform an ultrasound and CT scan of the abdomen in patients with otherwise unexplainable abdominal problems. In uncertain clinical cases, we should always perform a biopsy from the suspected resistance. In rare cases, the incarcerated ventral hernia could be the first manifestation of the sarcoma.
